# Safety and effectiveness of vagus nerve stimulation in patients with drug-resistant epilepsy: a single center experience

**DOI:** 10.1186/s42494-025-00215-5

**Published:** 2025-05-09

**Authors:** Ebtehal Alwazna, Jamal Abdullah, Hanin Alsini, Marahib Alshahrani, Wafa Aldhafeeri, Alawi Al-Attas, Abeer Alshaikh, Mashael Alanazi, Hamoud Alsahli, Mohammed Alshahrani, Shatha Alshafi, Brahim Tabarki, Abdulrahman Nazer, Sonia Khan

**Affiliations:** 1 Department of Neurology, Division of Epilepsy, Prince Sultan Military Medical City, Riyadh P. O. Box: 59046, Riyadh, 11159 Saudi Arabia; 2 Department of Neurosurgery, Prince Sultan Military Medical City, 11159 Riyadh, Saudi Arabia; 3 Department of Pediatrics, Division of Pediatric Neurology, Prince Sultan Military Medical City, Riyadh, Saudi Arabia; 4 Department of Neurology, King Saud Medical City, 12746, Riyadh, Saudi Arabia

**Keywords:** Drug-resistant epilepsy, Effectiveness, Interictal epileptiform discharges, Long-term outcomes, Quality of life, Safety profile, Seizure

## Abstract

**Background:**

Drug-resistant epilepsy (DRE) exerts substantial clinical, humanistic and economic burdens on patients, their families and the healthcare system. Vagus nerve stimulation (VNS) has been extensively tested in clinical trial settings to decrease the frequency of seizures in patients with DRE who are not candidates for surgery; the results indicate promising efficacy and a well-tolerated safety profile. However, real-world evidence is still lacking. This retrospective study evaluated the safety and efficacy of VNS in patients with DRE.

**Methods:**

The current study was a retrospective chart review of the medical records of children and adults with DRE treated with VNS between December 2006 and November 2022. The primary outcome of the present study was the percentage of patients who experienced a reduction in seizure frequency of more than 50% compared with the frequency at baseline (the period before VNS device insertion).

**Results:**

A total of 103 patients were included. The percentage of patients who achieved a reduction of more than 50% in seizure frequency was 23% at six months, 36% at 12 months, 65% at 18 months, and 72% at 24 months. Similarly, the percentage of patients with complete resolution of interictal epileptiform discharges (IEDs) increased from 30% at six months to 60% after 24 months. The overall Quality of Life in Epilepsy (QOLIE-31) score at the end of follow-up was 39.46 ± 13.68 points. Two patients (1.9%) reported experiencing side effects at the end of follow-up.

**Conclusions:**

VNS implementation led to a significant reduction in the seizure frequency and resolution of IEDs, with a well-tolerated safety profile. The findings highlight the potential role of VNS in managing DRE and warrant its consideration for treating patients with DRE.

**Supplementary Information:**

The online version contains supplementary material available at 10.1186/s42494-025-00215-5.

## Background

Epilepsy is a well-known chronic neurological disorder characterized by recurrent seizures, affecting almost 70 million patients worldwide [1]. Among those diagnosed, a significant portion—approximately 30%—suffered from drug-resistant epilepsy (DRE), defined as failure to achieve seizure freedom despite adequately choosing two or more antiseizure medication trials [2, 3]. Patients with DRE are at increased risk of sudden unexplained death due to epilepsy, injury, cognitive decline, and psychiatric comorbidities. Moreover, social and psychological impacts, such as stigmatization, unemployment, and reduced quality of life (QoL), significantly burden patients and their families [4]. In response to these challenges, various alternatives to improve outcomes for patients with DRE include etiology-specific drugs, surgical interventions, ketogenic diets, and neuromodulatory therapies such as vagus nerve stimulation (VNS) [5].

VNS, a neuromodulator, has become an important treatment for drug-resistant epilepsy (DRE) to reduce the frequency and severity of seizures [6]. In 1997, the FDA approved VNS for epilepsy treatment, demonstrating an improvement in the quality of life of patients unresponsive to conventional medication therapies [7].

Despite the growing interest in VNS as a therapeutic intervention, the literature presents a mixed understanding of its long-term efficacy and safety. Although several studies have reported positive outcomes, including significant reductions in seizure frequency and enhanced quality of life, gaps in knowledge regarding specific patient populations and factors that may influence treatment success remain uncertain [8–11]. The VNS therapy system has undergone significant advancements since its inception [12]. Initially, the Pulse™ M102, released in 2002, introduced a programable pulse generator, lead, and external system for adjusting the stimulation settings. Subsequent innovations included the Demipulse® M103 in 2007 and the smaller Demipulse Duo® M104 in 2011, which featured updated diagnostic tools [13]. The AspireHC® M105, introduced later in 2011, offered a larger design with a high-capacity battery, providing a 36% longer lifespan compared to earlier models [14]. The AspireSR® M106, launched in 2015, marked a pivotal advancement with its responsive, closed-loop system capable of detecting and responding to rapid heart rate changes indicative of seizures [15]. The AutoStim mode enhances the therapeutic efficacy in pediatric and adult patients with diverse epilepsy types [16, 17].

This retrospective study assessed the safety and efficacy of VNS in patients with DRE with the aim of improving understanding and providing valuable insight into the use of VNS as a therapeutic option for DRE.

## Methods

### Aim, design, and setting

The aim of this study was to evaluate the safety and efficacy of VNS in patients with DRE. The study was designed as an open-label, uncontrolled, retrospective chart review. The study was conducted at the Epilepsy Center of Prince Sultan Military Medical City (PSMMC) in Riyadh, Saudi Arabia.

### Participants

The study included 103 pediatric and adult patients diagnosed with DRE, defined as persistent seizures despite treatment with at least two different syndrome-ASMs. All participants underwent VNS device implantation between December 2006 and November 2022. The patients had undergone extensive presurgical evaluations, including video-EEG monitoring, neuroimaging, and neuropsychological assessment, and were deemed unsuitable for epilepsy surgery. Only patients with a minimum of 12 months of follow-up data after VNS implantation were included in the study. The exclusion criteria were incomplete medical records, concurrent neurostimulation therapies, coexisting major neurological disorders (e.g., progressive neurodegenerative diseases), and major surgery or trauma during follow-up.

The initial device settings were a current of 0.25 mA, a frequency of 30 Hz, and a pulse width of 250 μsec, with a 30 s “on-time” and 5.0 min “off-time”. Device settings were gradually adjusted based on patient tolerance and response to therapy. Regular monitoring of the treatment efficacy and adverse effects was performed throughout the study.

### Data collection and study outcomes

The following data were collected from the medical records of eligible patients: age, sex, family history of epilepsy, epilepsy etiology, epilepsy type and semiology, seizure duration before device implantation, VNS device model, date of VNS device implantation, device settings, follow-up duration, seizure frequency during the follow-up period, changes in seizure severity, frequency of interictal epileptiform discharges (IEDs), changes in the ASM regimen, QoL as assessed by the Quality of Life in Epilepsy (QOLIE-31) and reported side effects or complications associated with VNS therapy [18].

### Statistical analysis

The data were analyzed using the Statistical Package for the Social Sciences (SPSS) software (version 28.0). Descriptive analysis was employed, with the mean ± standard deviation (SD) reported for numerical data and the number (percentage) reported for categorical data. The primary outcome, a > 50% reduction in the seizure frequency after VNS device implantation, was assessed using a binomial proportion test with confidence intervals. The associations between the primary outcomes and participant characteristics were evaluated using chi-square tests, Fisher’s exact tests, paired *t*-tests, or nonparametric equivalent tests, as appropriate. A significance level of *P* < 0.05 was set for all tests.

## Results

The study included 103 patients. Of the included patients, 55.3% were male and 77.7% were older than 20 years. The median age at seizure onset was 4 years, and the median age at VNS device implantation was 21 years. Fourteen percent had an abnormal perinatal history, 15% had a family history of epilepsy, and 42.7% had a history of abnormal development.

The causes of epilepsy varied among the patients, with 39.7% having structural brain causes, 29.1% having unknown causes, and 13.6% having genetic abnormalities. The most common seizure type was generalized tonic–clonic seizures (92.2%), followed by symmetric tonic seizures (2.9%) and myoclonic seizures (1.9%).

Before the implantation of the VNS device, the mean seizure duration was 1.85 ± 1.35 min, and the mean seizure frequency was 6.94 ± 7.33 attacks per week (Table 1).

**Table 1:** Demographic and clinical characteristics of patients before VNS device implantation

Characteristics		Patients (*n* = 103)	
		**Frequency**	**%**
**Male sex**		57	55.3
**Age group**	< 10 years	2	1.9
	10–20 years	21	20.4
	21–30 years	28	27.2
	31–40 years	36	35.0
	41–50 years	10	9.7
	> 50 years	6	5.8
**Nationality**	Saudi	98	95.1
	Non-Saudi	5	4.9
**Abnormal perinatal history**		14	13.6
**Family history of epilepsy**		15	14.6
**Abnormal development**		44	42.7
**Etiology**	Genetic	14	13.6
	Structural	39	37.9
	Posttraumatic	8	7.8
	Postinfectious	3	2.9
	Unknown etiology	30	29.1
	Others	9	8.7
**Seizure type**	Generalized epilepsy	102	99.0
	Focal epilepsy	1	1.0
**Seizure semiology**	Generalized tonic–clonic seizures	95	92.2
	Symmetric tonic seizures	3	2.9
	Myoclonic seizure	2	1.9
**Seizure duration (min), mean ± SD**		1.9 ± 1.4	
**Seizure frequency, mean ± SD**		6.9 ± 7.3	

**Fig. 1:**
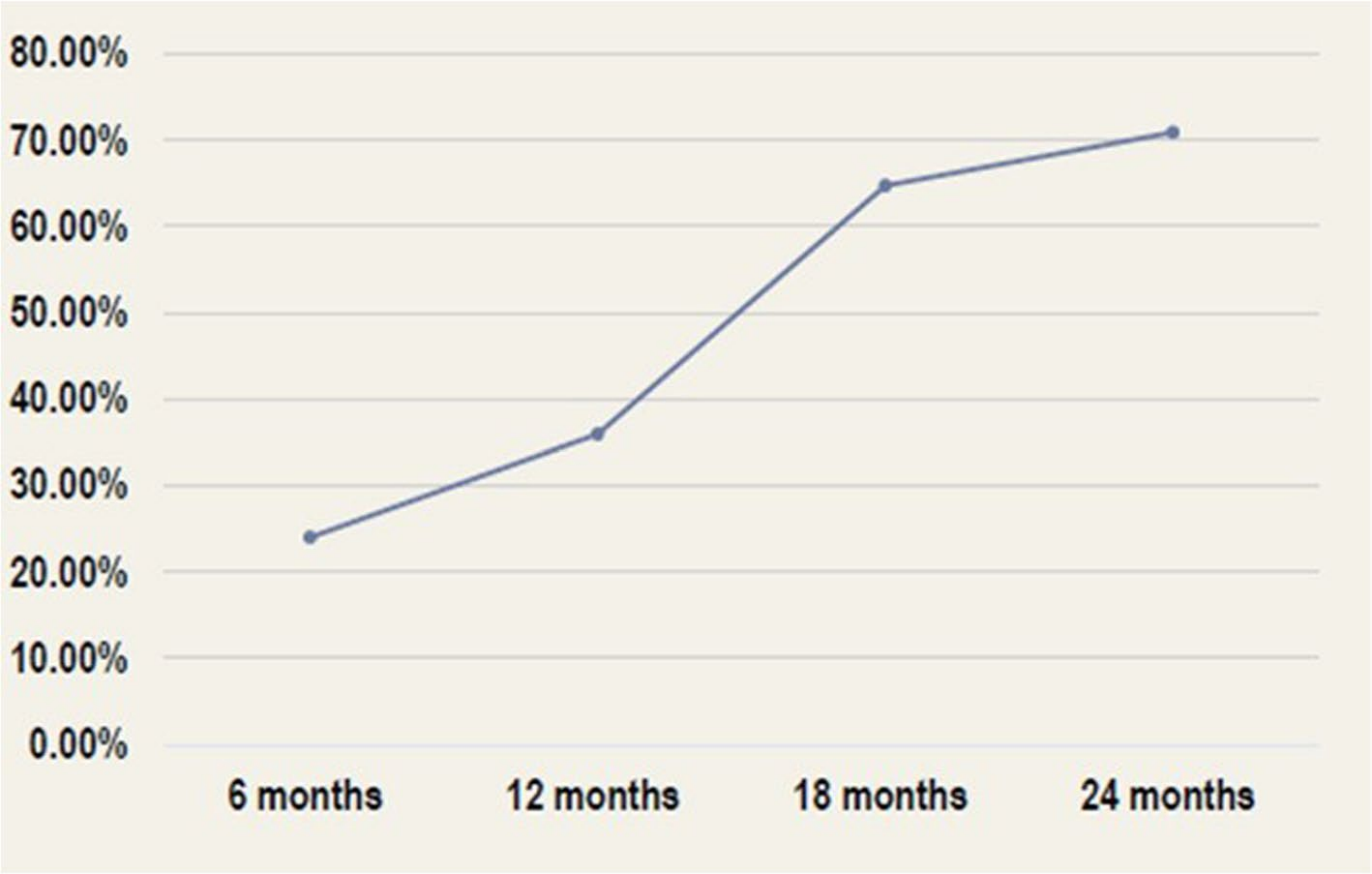
Percentage of patients who achieved a 50% reduction in seizure frequency after VNS device implantation

**Fig. 2:**
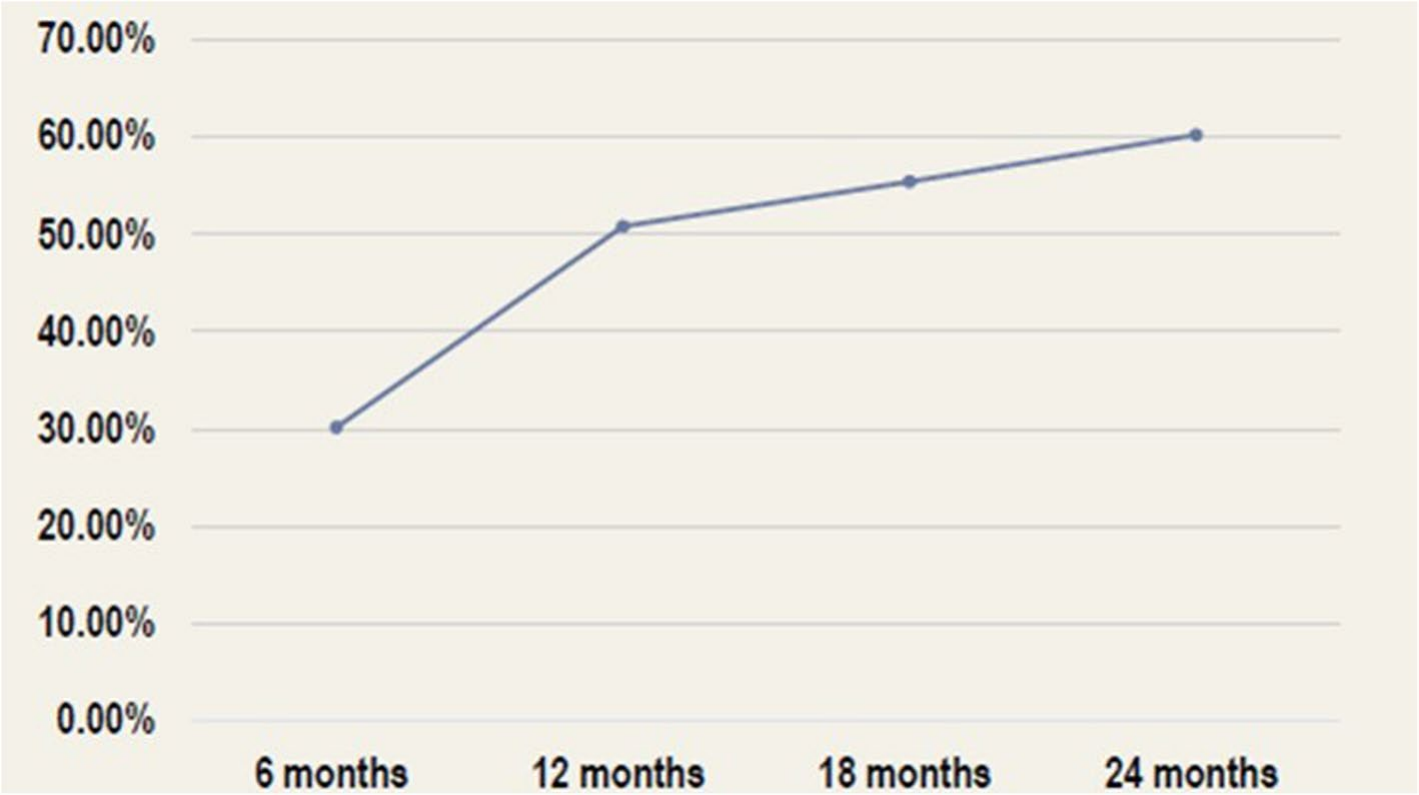
Percentage of patients with IED resolution after VNS device implantation

**Fig. 3:**
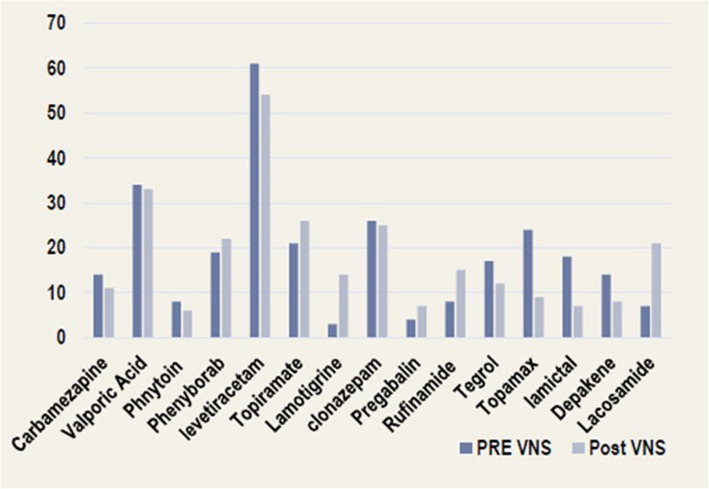
Number of ASMs taken by patients before VNS device insertion and at the last follow-up visit

A total of 98 patients were followed up for at least 24 months after VNS device implantation. The proportion of patients who experienced a > 50% reduction in seizure frequency was 23% at six months, 36% at 12 months, 65% at 18 months, and 72% at 24 months (Fig. 1). Concerning secondary outcomes, the proportion of patients with complete resolution of IEDs increased from 30% at six months to 51% at 12 months, 56% at 18 months, and 60% at 24 months (Fig. 2). At the time of VNS device implantation, the average number of ASMs per patient was 3.6. No new ASMs were added during the peri-implantation period. At the last follow-up, the average number of ASMs prescribed per patient decreased slightly to 2.2. However, this decline was not statistically significant (Fig. 3). The overall QOLIE-31 score at follow-up was 39.46 ± 13.68 points. Two patients (1.9%) reported experiencing side effects at the end of follow-up.

The association analysis revealed no significant associations between epilepsy etiology and a > 50% reduction in seizure frequency (*P* = 0.24), complete resolution of IEDs (*P* = 0.58), or the QOLIE-31 total score (*P* = 0.383), as shown in Supplementary Tables S1 and S2. Similarly, no significant associations were found between age at onset and a > 50% reduction in seizure frequency (*P* = 0.50), complete resolution of IEDs (*P* = 0.069), or the QOLIE-31 cognitive subdomain score (*P* = 0.82), as demonstrated in Supplementary Table S3.

Moreover, the analysis indicated that the treatment response (*P* = 0.63) and EEG improvement (*P* = 0.78) did not differ significantly across various VNS system configurations. However, there was a significant difference in the cognitive outcomes and overall QoL, as measured by the QOLIE-31. The mean QOL T-score was 39.46 (SD = 13.68). Patients who received a VNS system with configurations of 103/106 were more likely to have average cognitive scores at follow-up (*P* = 0.011). Similarly, patients with a VNS system with configurations of 102/106 had significantly higher QOLIE-31 scores (*P* = 0.008) (Table 2).

**Table 2:** Association between the VNS system used and the treatment outcomes

		**VNS System**														***P* value**
		102	102/102/105	102/103	102/103/105	102/103/106/106	102/105	102/106	102/106/106	102\103	103	103/106	103\106\106	106	106/106	
**Seizure Response, No**	< 50 (*n* = 30)	5.00	0.00	1.00	0.00	0.00	0.00	1.00	0.00	0.00	6.00	4.00	0.00	13.00	0.00	0.63
	> 50 (*n* = 73)	17.00	1.00	4.00	1.00	1.00	1.00	8.00	2.00	1.00	9.00	4.00	1.00	20.00	3.00	
**EEG improvement, No**	No improvement (*n* = 41)	7.00	0.00	1.00	0.00	0.00	0.00	3.00	1.00	0.00	7.00	5.00	0.00	15.00	2.00	0.78
	Resolution of IEDs (*n* = 62)	15.00	1.00	4.00	1.00	1.00	1.00	6.00	1.00	1.00	8.00	3.00	1.00	18.00	1.00	
**QOLIE-31 cognitive subdomain score, No**	Below average (*n* = 3)	0.00	0.00	1.00	0.00	0.00	0.00	1.00	1.00	0.00	0.00	0.00	0.00	0.00	0.00	0.01
	Borderline (*n* = 9)	4.00	1.00	1.00	1.00	0.00	0.00	2.00	0.00	0.00	0.00	0.00	0.00	0.00	0.00	
	Low (*n* = 3)	0.00	0.00	1.00	0.00	0.00	0.00	1.00	1.00	0.00	0.00	0.00	0.00	0.00	0.00	
	Mild (*n* = 2)	0.00	0.00	0.00	0.00	0.00	0.00	0.00	0.00	0.00	0.00	0.00	0.00	2.00	0.00	
	Normal (*n* = 85)	17.00	0.00	2.00	0.00	1.00	1.00	5.00	0.00	1.00	15.00	8.00	1.00	31.00	3.00	
	Poor (*n* = 1)	1.00	0.00	0.00	0.00	0.00	0.00	0.00	0.00	0.00	0.00	0.00	0.00	0.00	0.00	
**QOLIE-31 total score, mean ± SD**		40.36 ± 9.90	62.00	54.00	69.00	59.00	NR	49.29 ± 17.40	64.00	37.00	39.30 ± 16.60	32.00	18.00	35.04 ± 10.20	38.00 ± 7.90	0.008

In this study, we observed a minimal incidence of side effects associated with VNS, which hindered treatment continuation. Specifically, only two patients experienced significant adverse effects that necessitated discontinuation of the therapy. Among these patients, one reported an inability to tolerate the device, leading to a request for its removal. The low incidence of side effects underscores the overall safety and tolerability of VNS for managing drug-resistant epilepsy in our cohort.

## Discussion

We evaluated the outcomes of adult and pediatric patients with DRE after VNS implantation for 16 years. This study involved a previously unstudied patient group comprising individuals of various ages, epilepsy types, and comorbid health conditions, with a long follow-up period to evaluate the long-term safety and efficacy of the treatment. At 24 months after implantation, 72% of the patients achieved a > 50% reduction in seizure frequency. In line with our findings, a recent retrospective study of 95 patients with DRE due to structural brain damage showed a > 50% seizure reduction in 60% of patients, with a 10% seizure-free rate at 24 months of implantation [19]. Another study on patients with DRE associated with the tuberous sclerosis complex reported that 70.6% achieved a > 50% reduction in seizure frequency after a mean follow-up period of 4.1 years [20]. Long-term follow-up (≥ 2 years) demonstrated a 76% response rate to VNS, with 33% of the patients reporting no incidents of the most disabling seizure type [21]. Similarly, a recent critical review of studies evaluating VNS over 30 years found that the response rate (> 50% reduction in seizure frequency) ranged from 45 to 65% [22]. In addition, 60% of the patients achieved complete resolution of the IEDs by the 24-month follow-up. Although the average number of ASMs per patient showed a slight reduction at the end of follow-up, no patient was fit to discontinue all ASMs, reflecting an improvement in seizure control and a decrease in epilepsy severity.

Identifying responders to VNS has proven challenging, given the variability in the presumed etiologies of epilepsy, like genetic diseases, structural brain lesions, post-traumatic brain injury, post-CNS infection, and other unknown causes, as well as differences in age of onset, seizure type, and VNS model. Because our study mostly reported a generalized seizure type, this may indicate a favorable response to VNS therapy.

The responders in our study (patients with > 50% seizure reduction) showed gradual improvement with follow-up visits. The percentage of patients was 23% at 6 months, 36% at 12 months, 65% at 18 months, and 72% at 24 months. According to studies evaluating the efficacy over time, the response rate increased from 40% at 1 year to 60% at 24 months post-treatment [23]. Additionally, it is worth mentioning that a meta-analysis of VNS responder rates across 78 studies (*n* = 2869 patients) revealed a notable increase from year 2 to year 5 post-implantation [24]. Although cumulative evidence indicates that the effectiveness of VNS evolves over time in patients with DRE, the exact mechanisms underlying its antiseizure effects remain unclear and multifaceted. One primary theory is the modulation of neurotransmitter systems; specifically, the VNS increases inhibitory neurotransmitters like GABA and reduces excitatory neurotransmitters such as glutamate, thereby altering neuronal excitability [25].

Moreover, VNS stimulation disrupts the abnormal synchronization of neuronal activity characteristic of seizures, thereby contributing to its seizure-reducing effect [26]. Additionally, VNS modulates brainstem centers responsible for cortical activity, which may play a role in its anticonvulsant effects. Current evidence suggests that the neurotrophic effects of the VNS potentially promote neuroplastic changes that contribute to long-term seizure reduction [25, 26]. Notably, emerging research has indicated that the VNS may exert anti-inflammatory effects in the brain, which is relevant given the proposed role of inflammation in epilepsy [5].

VNS models progressed throughout this study, with multiple VNS models implanted in our patients, including an automatic stimulation mode (AutoStim) that stimulates the vagus nerve upon detecting tachycardia. The AutoStim mode increases the therapeutic efficacy of VNS therapy in both pediatric and adult patients with various epilepsy etiologies [16, 17]. Patients with previous VNS models were successfully replaced with the Autostim model, resulting in overlapping outcomes that required careful evaluation during analysis.

Seizure freedom is widely recognized as the most significant predictor of quality of life (QOL) in patients with epilepsy [27]. The quality of life in the epilepsy inventory-31 (QOLIE-31) scoring system was applied to assess QOL. It contains seven multi-item scales and keys to convert the raw numeric value to a point score and to determine the total score, which represents linear transformations of the scores that produce a mean of 50 and standard deviation (SD) of 10 for the cohort of 304 adults with epilepsy. Thus, a person with a T-score of 50 equals the mean score of the epilepsy cohort [18]. In this study, the mean QOL T-score results were 39.46 (SD:13.68).

A surgical procedure is necessary to implant the VNS device, which involves typical surgical risks but is generally safe [28]. Common side effects include hoarseness, throat discomfort, and coughing during stimulation, and these are often managed by adjusting the device settings [28, 29]. In this study, VNS device implantation was well tolerated and did not lead to serious adverse events. The rate of adverse events was low, and the observed events were manageable. In agreement with our findings, a 25-year chart review of VNS procedures in a single center demonstrated that the procedure was well tolerated and that the rate of complications was low (2%) [30]. Similarly, a recent systematic review reported a low rate of adverse events following VNS device implantation, with no severe or life-threatening adverse events documented [28, 31]. Notably, VNS is also safe for children aged < 12 years, with outcomes comparable to those in older children and adults [32, 33].

This study is one of the few reports to evaluate the safety and efficacy of VNS therapy for individuals with DRE in Saudi Arabia. Applying standard measures for quality of life, such as QOLIE-31, enhances the value of our study; however, we acknowledge some limitations. As a retrospective chart review, this study may be subject to recall and misclassification bias. The lack of prospective follow-up data limited the ability to control for potential confounding variables. Additionally, the single-center nature of the study and the relatively small sample size may have affected the statistical power and generalizability of the results. Further studies are needed to identify patient-specific characteristics that may influence VNS efficacy.

## Conclusions

This study demonstrates the safety and effectiveness of VNS therapy in managing DREs. In our experience, VNS implementation led to a significant reduction in the seizure frequency and resolution of IEDs, with a well-tolerated safety profile. In addition, patients reported notable improvement in self-reported QoL. These findings highlight the potential role of VNS as a valuable treatment option for DRE and warrant its consideration for treating the patient population. Future studies with larger sample sizes and longer follow-up periods are needed to validate these results and further elucidate the mechanisms underlying the antiseizure effects of VNS.

## Supplementary Information

Supplementary Material 1.

## Data Availability

The data supporting the findings of this study are available from the corresponding author upon reasonable request.

## Abbreviations

ASM: Antiseizure medication
DRE: Drug-resistant epilepsy
EEG: Electroencephalogram
GABA: Gamma-aminobutyric acid
IED: Interictal epileptiform discharge
NCP: Neurocybernetic prosthesis
PSMMC: Prince Sultan Military Medical City
QoL: Quality of life
QOLIE-31: Quality of life in the epilepsy inventory
RCT: Randomized controlled trial
SD: Standard deviation
SPSS: Statistical Package for the Social Sciences
VNS: Vagus nerve stimulation
