# Supplementary material for: Safety and effectiveness of vagus nerve stimulation in patients with drug-resistant epilepsy: a single center experience
Source: Acta Epileptol. 2025 May 9;7:28. doi: 10.1186/s42494-025-00215-5 (PMC12063379; doi:10.1186/s42494-025-00215-5)
Supplement: Supplementary file 1 — Supplementary Material 1. [file 42494_2025_215_MOESM1_ESM.docx]

**Supplementary Tables**

Table S1: Association between Seizure Etiology and Outcomes: Seizure and EEG Response

| **Presumed Epilepsy Etiology** | **Outcomes. Seizure Response (<50% reduction in frequency/duration**  **= 0, >50% reduction**  **= 1)** | | **P value** |
| --- | --- | --- | --- |
|  | **<50** | **>50** |  |
| Anoxia | 2 | 7 | 0.24 |
| Arachnoid cyst | 0 | 1 |  |
| Bilateral perisylvian syndrome | 0 | 1 |  |
| CNS infection | 1 | 2 |  |
| Congenital hydrocephalus | 1 | 0 |  |
| FCD | 3 | 5 |  |
| Frontal lobe epilepsy | 1 | 1 |  |
| GBS variant | 1 | 0 |  |
| Genetic | 5 | 8 |  |
| Gliosis, and generalized brain atrophy | 0 | 1 |  |
| Hippocampal sclerosis | 0 | 1 |  |
| Hydrocephalus | 0 | 1 |  |
| Left hemispheric epilepsy | 1 | 0 |  |
| Lissencephaly | 1 | 1 |  |
| Mammillary body | 0 | 1 |  |
| Middle cerebral artery old infarction | 0 | 1 |  |
| MTS | 1 | 3 |  |
| MTS & FCD | 1 | 0 |  |
| NF | 0 | 1 |  |
| PME | 1 | 2 |  |
| PMG | 3 | 0 |  |
| PTE | 2 | 6 |  |
| Schizencephaly | 1 | 0 |  |
| TSC | 1 | 4 |  |
| Unknown | 4 | 26 |  |
|  | | | |
| **Presumed Epilepsy Etiology** | **Outcome. EEG Response (no improvement=0,**  **resolution of IEDs=1)** | | **P**  **value** |
|  | **No improvement** | **Resolution of IED** |  |
| Anoxia | 4 | 5 | 0.58 |
| Arachnoid cyst | 0 | 1 |  |
| Bilateral perisylvian syndrome | 0 | 1 |  |
| CNS infection | 1 | 2 |  |
| Congenital hydrocephalus | 1 | 0 |  |
| FCD | 2 | 6 |  |
| Frontal lobe epilepsy | 1 | 1 |  |
| GBS variant | 1 | 0 |  |
| Genetic | 7 | 6 |  |
| Gliosis, and generalized brain atrophy | 0 | 1 |  |
| Hippocampal  sclerosis | 0 | 1 |  |
| Hydrocephalus | 0 | 1 |  |
| Left hemispheric epilepsy | 1 | 0 |  |
| Lissencephaly | 1 | 1 |  |
| Mammillary body | 0 | 1 |  |
| Middle cerebral artery old infarction | 0 | 1 |  |
| MTS | 1 | 3 |  |
| MTS & FCD | 1 | 0 |  |
| NF | 0 | 1 |  |
| PME | 1 | 2 |  |
| PMG | 3 | 0 |  |
| PTE | 3 | 5 |  |
| Schizencephaly | 1 | 0 |  |
| TSC | 3 | 2 |  |
| Unknown | 9 | 21 |  |

*Abbreviations: IED Interictal discharges, FCD Focal cortical dysplasia, GBS Guillain-Barré syndrome, MTS Mesial temporal sclerosis, NF Neurofibromatosis, PME Progressive myoclonus epilepsy, PGE Polymicrogyria, PTE Post-traumatic epilepsy, TSC Tuberous sclerosis complex*

Table S2: Association between seizure etiology and outcome (QOL)

| **Overall** | | | | **P value** |
| --- | --- | --- | --- | --- |
| **Presumed Epilepsy Etiology** | **Mean** | **Number** | **STD** |  |
| Anoxia | 37.80 | 5 | 18.116 | 0.383 |
| CNS infection | 30.67 | 3 | 11.150 |  |
| Congenital  hydrocephalus | 37.00 | 1 | - |  |
| FCD | 45.25 | 4 | 17.289 |  |
| Frontal lobe epilepsy | 35.00 | 2 | 7.071 |  |
| GBS variant | 40.00 | 1 | - |  |
| Genetic | 29.22 | 9 | 10.402 |  |
| Gliosis, and generalized brain atrophy | 48.00 | 1 | - |  |
| Hydrocephalus | 33.00 | 1 | - |  |
| Left hemispheric epilepsy | 33.00 | 1 | - |  |
| Lissencephaly | 26.00 | 1 | - |  |
| Mammillary body | 44.00 | 1 | - |  |
| MTS | 53.75 | 4 | 16.091 |  |
| MTS & FCD | 41.00 | 1 | - |  |
| PME | 36.67 | 3 | 9.504 |  |
| PMG | 34.67 | 3 | 12.583 |  |
| PTE | 54.75 | 4 | 14.056 |  |
| Schizencephaly | 32.00 | 1 | - |  |
| TSC | 46.67 | 3 | 20.817 |  |
| Unknown | 40.24 | 21 | 12.280 |  |
| Total | 39.46 | 70 | 13.685 |  |

*Abbreviations: QOL Quality of life, STD Standard deviation, FCD Focal cortical dysplasia, GBS Guillain-Barré syndrome, MTS Mesial temporal sclerosis, NF Neurofibromatosis, PME Progressive myoclonus epilepsy, PGE Polymicrogyria, PTE Post-traumatic epilepsy, TSC Tuberous sclerosis complex*

Table S3: Association between Age of Seizure Onset and Outcomes: Seizure, EEG, and Cognitive Responses

| **Seizure onset (age in years)** | | | | |
| --- | --- | --- | --- | --- |
| **Outcome. Seizure**  **Response (<50%**  **reduction in**  **frequency/duration**  **= 0, >50% reduction**  **= 1)** | **Mean** | **Number** | **STD** | **P value** |
| <50 | 5.5967 | 30 | 6.68635 | 0.50 |
| >50 | 6.5492 | 73 | 6.42760 |  |
| Total | 6.2717 | 103 | 6.48559 |  |
|  | | | | |
| **Outcome. EEG Response (no improvement=0, resolution of IEDs=1)** | **Mean** | **Number** | **STD** | **P value** |
| No improvement | 4.8463 | 41 | 6.27551 | 0.069 |
| Resolution of IED | 7.2144 | 62 | 6.49935 |  |
| Total | 6.2717 | 103 | 6.48559 |  |
|  | | | | |
| **Outcome. IQ or cognitive sum score** | **Mean** | **Number** | **STD** | **P value** |
| Below average | 6.3333 | 3 | 7.76745 | 0.82 |
| Borderline | 5.3533 | 9 | 4.55424 |  |
| Low | 5.2000 | 3 | 7.62365 |  |
| Mild | 1.4000 | 2 | 0.84853 |  |
| Normal | 6.5825 | 85 | 6.71820 |  |
| Poor | 0.9000 | 1 | - |  |
| Total | 6.2717 | 103 | 6.48559 |  |

*Abbreviations: STD Standard deviation, IED Interictal discharges*
